# In Reply: Antibiotic-Impregnated Ventriculoperitoneal Shunts Decrease Bacterial Shunt Infection: A Systematic Review and Meta-Analysis

**DOI:** 10.1227/neu.0000000000003154

**Published:** 2024-08-30

**Authors:** Janka Kovács, Vanda Máté, Mahmoud Obeidat, Rita Nagy, Gergely Agócs, Szilvia Kiss-Dala, Péter Hegyi, Renáta Kiss-Miki, Andrea Párniczky, Katalin E. Müller, Miklós Garami

**Affiliations:** * Centre for Translational Medicine, Semmelweis University, Budapest, Hungary; ‡ Pediatric Center, MTA Center of Excellence, Semmelweis University, Budapest, Hungary; § Heim Pál National Pediatric Institute, Budapest, Hungary; ‖ Institute for Translational Medicine, Medical School, University of Pécs, Pécs, Hungary; ¶ Department of Biophysics and Radiation Biology, Semmelweis University, Budapest, Hungary; # Institute of Pancreatic Diseases, Semmelweis University, Budapest, Hungary; ** Department of Family Care Methodology, Faculty of Health Sciences, Semmelweis University, Budapest, Hungary

To the Editor:

We appreciate the opportunity to respond to the letter by Dr Victor M. Lu^1^ regarding our recent publication on the efficacy of antibiotic-impregnated ventriculoperitoneal shunts (AISCs).^2^ We value the scrutiny and acknowledge the points raised, which highlight the complexity of synthesizing research across diverse clinical scenarios. Below, we address each concern to clarify our methodology and findings.

**FIGURE 1.**
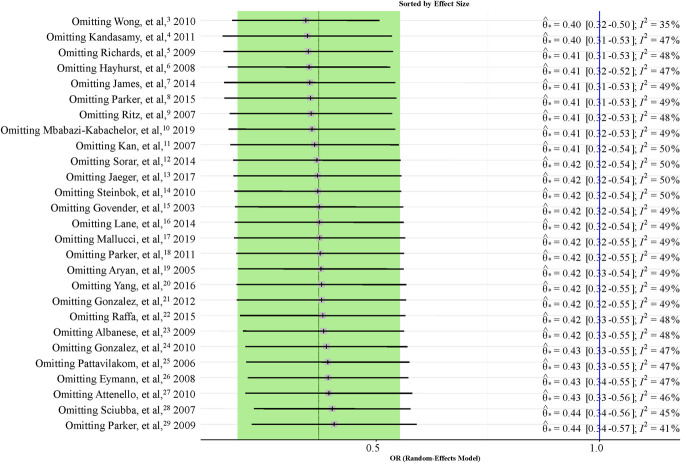
Funnel plot of random-effects model.^3-29^

**FIGURE 2.**
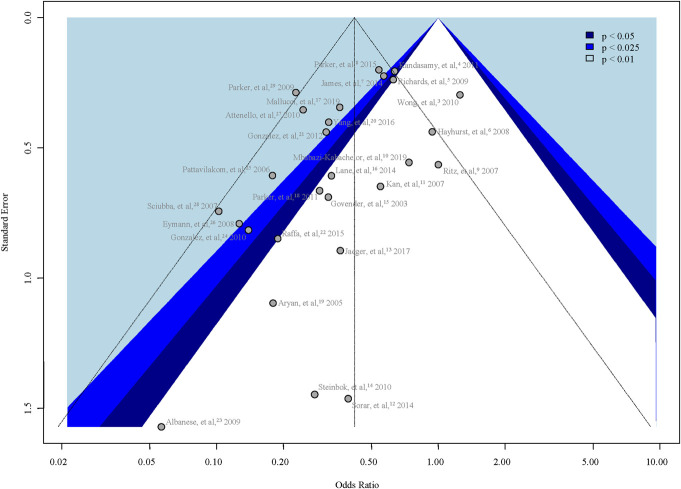
Leave-one-out analysis.^3-29^

1. Generalizability and Etiologies: Dr Lu's concern about the generalizability of our results due to the inclusion of various etiologies is insightful. Our decision to include a broad range of etiologies was intentional and aimed at reflecting the diverse clinical situations in which AISCs are used. This intention of ours was further supported by the fact that all the included studies contained data for all etiologies, not just some selected ones. We believe that this broad approach enhances the clinical applicability of our findings. However, we acknowledge that different etiologies may respond differently to AISCs, a point that warrants further discussion and exploration in future studies. Regarding the statistical aspects of the generalizability of results, significance per se always characterizes the strength of evidence provided in the given study setting, so it is not generalizable as described by Dr Lu. What is generalizable is the pooled effect size (here: the odds ratio [OR]); however, we did not declare equivalence limits, and therefore, the lack of significance in some of our outcomes does not imply evidence for equivalence.
2. Age Group Analysis: We conducted subgroup analyses to specifically address the variability in outcomes between different age groups, including pediatric vs adult populations and infants below one year vs older patients. These analyses were designed to identify any differential impacts of AISCs across these groups, and we believe they strengthen our overall conclusions by highlighting where effects may differ significantly. You correctly highlighted that in some cases, the heterogeneity was significant; however, significant heterogeneity does not automatically imply that it is high as well; instead, it means that it is not attributable solely to chance. We disagree with the statement that “this would lower the certainty in the summarized outcomes reported even more” because we did not assume homogeneity during the statistical analysis; as described in the methodology, we used a random-effects model where the outcome's CI reflects the effect of heterogeneity as well.
3. Leave-One-Out Sensitivity Analysis for Parker et al: Dr Lu mentioned that the leave-one-out analysis for the Parker et al (2011) study could be insightful because it represents 50% of the total patient count of all included studies. We think he intended to refer to the Parker et al (2015) study, which indeed contains 12 589 of the total 27 265 patients represented in the overall primary outcome analysis. However, how strong an influence a study has is not determined by just sheer sample size, it is more important what the point estimate (here: OR = 0.54) and its error is. The former is quite close to the overall pooled effect size (OR = 0.42). The latter is related to but not equivalent with the sample size; in the forest plot, it is reflected in the confidence interval and, ultimately, in the study weight. The weight of Parker et al, 2015 is the highest of all included studies (7.9%), but it is far from extreme; 3 other studies have over 7% weight. Nevertheless, we performed leave-one-out sensitivity analysis (Figure 1.^3-29^) to demonstrate the effect of each study on the pooled effect size: leaving out Parker et al, (2015) yields an estimate of OR = 0.41 (0.31-0.53), so the influence is neither statistically significant nor practically relevant. We also provide the funnel plot (Figure 2.^3-29^): Parker et al, (2015) does not seem to have a strong contribution to plot asymmetry on visual assessment neither is the overall plot asymmetry significant (Peters test *P* = .1027).
4. On the Use of the Term “Tendency”: Dr Lu points out the semantical issues with the use of the term “tendency.” We agree that the term does not have a universally established statistical definition. We used it to refer to the summary effect size (point estimate) in case we judged it to be clinically relevant in magnitude. However, we provided CIs for all outcomes both numerically and visually so that readers can assess our results and judge our conclusions.

Furthermore, we would like to cite that article (Anand A., et al, 2024), where our meta-analysis was mentioned to prove the importance of meta-analyses about antibiotic-impregnated shunts.^30^

We thank Dr Lu for his critical review and constructive comments, which undoubtedly contribute to the ongoing dialog and development of best practices in the use of AISCs. We look forward to further discussions and research in this area to refine and enhance the application of these findings in clinical practice.
